# Electrochemical and Optical Properties of D-A-A-A-D Azomethine Triad and Its NIR-Active Polymer

**DOI:** 10.3390/molecules29184470

**Published:** 2024-09-20

**Authors:** Mateusz Roszyk, Monika Wałęsa-Chorab

**Affiliations:** Faculty of Chemistry, Adam Mickiewicz University in Poznań, Uniwersytetu Poznańskiego 8, 61-614 Poznań, Poland

**Keywords:** azomethine, electrochromism, luminescence, solvatochromism, electropolymerization

## Abstract

The azomethine **TPA-(BTZ)_3_-TPA** with a donor–acceptor–acceptor–acceptor–donor structure has been synthesized and characterized. Azomethine **TPA-(BTZ)_3_-TPA** exhibited luminescence properties and a positive solvatochromic effect. Electropolymerization on terminated triphenylamine groups was used to obtain a thin layer of the polyazomethine **poly-[TPA-(BTZ)_3_-TPA]**. Further investigation of oxidation/reduction properties of **poly-[TPA-(BTZ)_3_-TPA]** via cyclic voltammetry showed that the polymer undergoes two reversible oxidation/reduction processes due to the presence of tetraphenylbenzidine moieties. Electrochromic properties of the polyazomethine **poly-[TPA-(BTZ)_3_-TPA]** were investigated via spectroelectrochemistry. It was observed that the polymer in its neutral state is orange, and the color changes to green upon electro-oxidation. The stability of the polymer during multiple oxidation/reduction cycles, response times, and coloration efficiency were also investigated.

## 1. Introduction

Azomethines, which are secondary aldimines, are synthesized through the simple condensation of primary amines with carbonyl compounds, often carried out in mild reaction conditions [1]. The conjugation of multiple azomethine units leads to the formation of polyazomethines, also called poly (Schiff bases). Azomethines and polyazomethines present a viable alternative to vinylene-based optoelectronic materials due to their isoelectronic nature, simplified synthesis processes, and comparable properties [2,3]. However, their applications in plastic electronics are limited due to their low resistance to oxidation and hydrolysis [4,5]. On the other hand, the dynamic behavior of the conjugated azomethine bond can be an advantage allowing the adjustment of the properties of azomethines polymers [6,7,8]. Furthermore, organic materials derived from azomethines are cost-effective to produce, and the products are obtained with a high degree of purity and yield, because the only byproduct of their synthesis is water [9,10]. 

The formation of polyazomethines can be performed in two ways: (i) polycondensation of appropriate diamines and dialdehydes [11,12,13,14] or (ii) polymerization of azomethine monomers [8,15,16,17]. A polycondensation reaction is usually carried out in the presence of a catalytic amount of a Lewis acid (such as trifluoroacetic acid or scandium trifluoromethane sulfonate), and it can be accomplished via traditional methods in solution [11,12,13,14], with microwave-assisted synthesis [18] in different organic solvents, via hydrothermal polymerization [19], or with on-substrate polymerization [13,14,20]. The second method of formation of polyazomethines relies on the formation of azomethine containing different polymerizable functional groups following polymerization using different polymerization reactions.

Schiff bases, known for their diverse range of applications, can serve as catalysts (e.g., salen catalysts in the Jacobsen epoxidation), ligands in coordination chemistry, pharmaceuticals, and corrosion inhibitors [21,22,23]. Beyond these traditional uses, there has been an increasing interest in the application of azomethines in optoelectronic devices such as photovoltaic cells [24,25,26], organic light-emitting diodes (OLEDs) [27,28,29], and memory devices [30,31,32]. Additionally, these compounds display electrochromic properties, which are advantageous for applications in electrochromic devices, such as smart windows [15,20,33,34,35]. Such applications are possible due to the good electrical conductance of the azomethine C=N bond, what makes it a useful linker for the preparation of conjugated materials for electronic applications [36]. 

Conjugated azomethines, characterized by multiple imine groups and conjugated π-systems, exhibit promising luminescent properties, making them ideal candidates for optoelectronic applications [37,38,39]. Notable examples include polyazomethines and azomethine triads, the latter being formed through the condensation of two equivalents of amine with one equivalent of aldehyde [40]. Moreover, the non-polar structure of azomethines makes them an ideal candidate for materials exhibiting aggregation-induced emission (AIE) [41,42,43]. The emission of azomethines is sometimes limited by fluorescence quenching, hindering their application in optoelectronics [44]. This limitation can be mitigated through structural modifications of the azomethine, such as altering functional groups. For instance, fluorene-based azomethines exhibit high fluorescence quantum yields but undergo intersystem crossing to the triplet excited state [45]. 

Moreover, in addition to the previously mentioned properties, fluorescent azomethines can display solvatochromism, altering their color in response to solvent polarity [46,47]. Such behavior is often observed for azomethines with a donor–acceptor structure. Similarly as for other donor–acceptor compounds [48,49,50], donor–acceptor azomethines are characterized by the lower energy intermolecular charge transfer (ICT) state [51,52], which shows huge red-shifting emission when increasing the solvent polarity. 

The incorporation of strongly electron-accepting benzothiadiazole fragments, in addition to leading to the formation of a charge transfer band, results in a low HOMO–LUMO gap [53,54]. This results in red-shifting of absorption and emission absorption wavelengths [55]. Additionally, the introduction of a strong acceptor also affect the electrochemical properties of materials and causes an increase in the reduction potential of the compound [56]. It was also described that for series of molecules with different donor-to-acceptor ratios, with an increase in the acceptor contribution a decrease in oxidation potential was observed [57]. 

**Figure 1:**
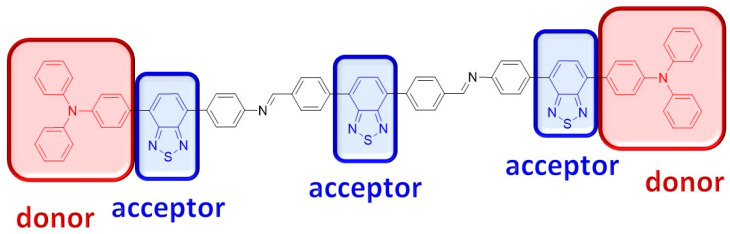
The structure of azomethine **TPA-(BTZ)_3_-TPA**.

In light of this, we designed and synthesized the azomethine **TPA-(BTZ)_3_-TPA** with a donor–acceptor–acceptor–acceptor–donor (D-A-A-A-D) structure (Figure 1) containing electron-donating triphenylamine groups and electron-accepting benzothiadiazole groups. The azomethine **TPA-(BTZ)_3_-TPA** was investigated in terms of its photophysical and electrochemical properties. It was found that the azomethine **TPA-(BTZ)_3_-TPA** electropolymerizes on the electrode surface, forming an orange electrochromic polyazomethine. The electrochemical and electrochromic properties of the polymer layer were also investigated. 

## 2. Results and Discussion

### 2.1. Synthesis of the Dye

**Figure 2:**
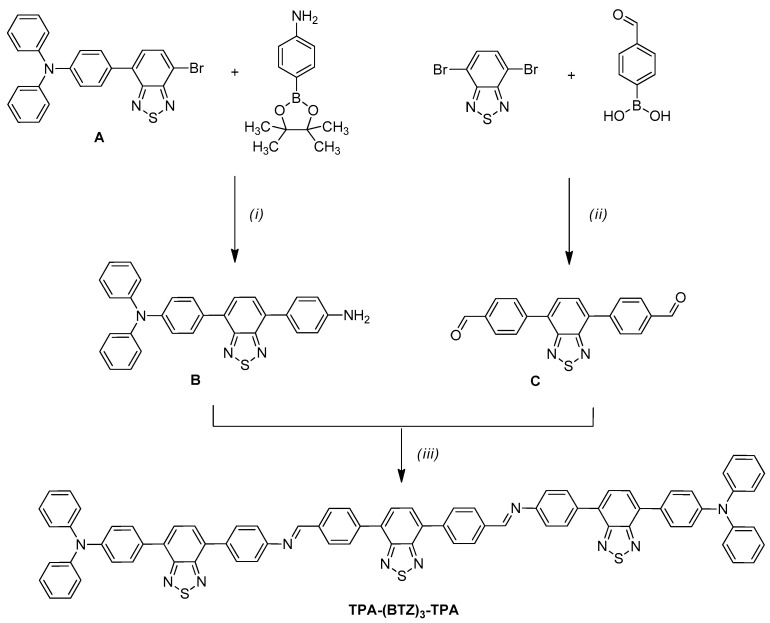
Synthetic scheme for the preparation of **TPA-(BTZ)_3_-TPA** azomethine: (i) toluene/water 2:1 *v*/*v*, Pd(PPh_3_)_4_, K_2_CO_3_, 90 °C, 24 h; (ii) toluene/water 2:1 *v*/*v*, Pd(PPh_3_)_4_, Na_2_CO_3_, TBABr, 95 °C, 24 h; (iii) THF, Sc(CF_3_SO_3_)_3_, RT, 24 h.

The synthesis of the conjugated azomethine **TPA-(BTZ)_3_-TPA** was carried out in three steps, with the synthesis scheme shown in Figure 2.

The initial reagent **A** has been obtained via a previously reported method [49], and it has been used in palladium-catalyzed Suzuki-Miyaura coupling reactions with an excess of 4-aminophenylboronic acid pinacol ester in the presence of base-potassium carbonate and tetrabutylammonium bromide as a phase transfer catalyst. The reaction was carried out in toluene/water mixture in 2:1 volume ratio for 24 h. The product **B** has been purified via column chromatography, and it has been obtained as a yellow solid with 60% yield. A similar protocol has been used to obtain dialdehyde **C** containing a benzothiadiazole core. In the final step, compound **B** was condensed with a dialdehyde **C** in the presence of scandium(III) trifluoromethanesulfonate as a Lewis acid. The precipitated product **TPA-(BTZ)_3_-TPA** was centrifuged and washed with hexane. At each step of the synthesis, the identity and purity of the products were confirmed using ^1^H NMR spectroscopy, ^13^C NMR spectroscopy, and mass spectrometry (Figures S1–S12). The formation of the targeted azomethine **TPA-(BTZ)_3_-TPA** was confirmed by the presence of a singlet at 8.67 ppm in the ^1^H NMR spectrum (Figure S1) originating from the hydrogen atoms of the imine bond (HC=N). Meanwhile, the singlet at 7.93 ppm is attributed to the hydrogen atoms of the central, symmetrically substituted benzothiadiazole group, while the doublets at 7.83 and 7.78 ppm originate from the hydrogen atoms of the two asymmetrically substituted benzothiadiazole groups. In the ^13^C NMR spectrum (Figure S2), 26 signals are visible, corresponding to the structure of the obtained compound. At 159.9 ppm, a peak characteristic for the imine carbon is visible, which also confirms the formation of the imine bond. The obtained compound was also characterized using mass spectrometry, as shown in Figure S3. In the spectrum, the molecular ion at *m*/*z* 1249.3605 is observed, which matches the calculated mass of the protonated compound (M+H)^+^. Furthermore, the observed isotopic distribution corresponds to the theoretical isotopic distribution for this structure. 

### 2.2. Optical Properties

**Figure 3:**
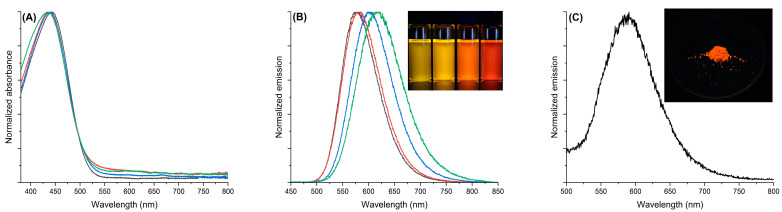
(**A**) Absorption spectra of azomethine **TPA-(BTZ)_3_-TPA** in toluene (black), dioxane (red), THF (blue), and dichloromethane (green); (**B**) normalized emission spectra of azomethine **TPA-(BTZ)_3_-TPA** in toluene (black), dioxane (red), THF (blue), and dichloromethane (green) when excited at the corresponding absorption maxima. Insert: photograph showing the fluorescence of azomethine **TPA-(BTZ)_3_-TPA** in toluene, dioxane, THF, and dichloromethane (from left to right) when excited with a handheld UV lamp (365 nm); (**C**) normalized emission of azomethine **TPA-(BTZ)_3_-TPA** in the solid state when excited at 430 nm. Insert: photograph showing the fluorescence of azomethine **TPA-(BTZ)_3_-TPA** in the solid state when excited with a handheld UV lamp (365 nm).

First, the obtained azomethine **TPA-(BTZ)_3_-TPA** was examined for its photophysical properties. The donor–acceptor–acceptor–acceptor–donor configuration of the molecule was expected to be sensitive to solvent polarity. Due to this, to investigate the solvatochromic properties of the dye, absorption and emission measurements were conducted in four solvents: toluene, tetrahydrofuran, dioxane, and dichloromethane, as shown in Figure 3. Non-normalized absorbance spectra are shown in Figure S13. Such solvents have been chosen due to their varying polarity and ability to solubilize the azomethine **TPA-(BTZ)_3_-TPA**. The related photophysical parameters are compiled in Table 1. As seen in Figure 3A, the compound exhibited the broad absorption bands in the visible range with the maxima at around 440 nm, which can be attributed to the intramolecular charge transfer (ICT) between donor and acceptor groups [58], and the absorption maxima were almost not affected by the solvent polarity. On the other hand, the emission wavelengths of azomethine **TPA-(BTZ)_3_-TPA** were found to be significantly shifted (by 41 nm) in different solvents, from 577 nm in non-polar toluene to 618 nm in polar dichloromethane (Figure 3B), indicating the existence of interactions between the dye and solvent molecules. Both compounds exhibited a positive solvatochromic effect. As seen in the picture inserted in Figure 3B, the color of emitted light changed from yellow in toluene to red in dichloromethane. 

The fluorescence quantum yield of the azomethine **TPA-(BTZ)_3_-TPA** was also evaluated in different solvents. This was accomplished using the integrating sphere calibrated using the calibrated light source, which allow the measurements of the absolute value of quantum yield and do not require the use of any standards. Taking into account the quenching character of the azomethine bond [59], the fluorescence quantum yield was found to be high. In toluene, the fluorescence quantum yield was measured to be 35%, and it decreased to 27% in dichloromethane. 

The azomethine **TPA-(BTZ)_3_-TPA** was also found to be emissive in the solid state. As seen in Figure 3C, when excited at 430 nm, the azomethine **TPA-(BTZ)_3_-TPA** emitted light with a maximum at 588 nm, and the emission of orange light was visible when irradiated with a handheld UV lamp (365 nm). The fluorescence quantum yield of the azomethine **TPA-(BTZ)_3_-TPA** in the solid state was measured to be 7%. 

### 2.3. Electropolymerization and Characterization

**Figure 4:**
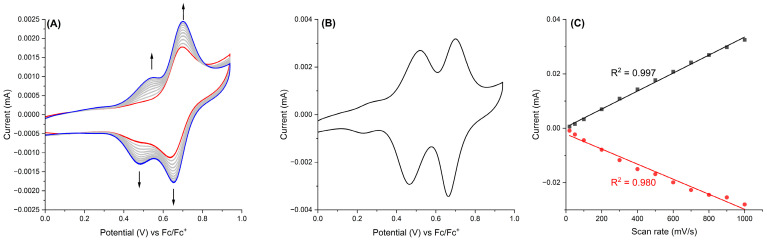
(**A**) Cyclic voltammograms recorded during electropolymerization of the azomethine triad **TPA-(BTZ)_3_-TPA** measured at room temperature in 0.1 M solution of TBAPF_6_ in dichloromethane as a supporting electrolyte on the Pt working electrode at a 100 mV/s scan rate; (red) 1st cycle, (blue) 10th cycle; (**B**) cyclic voltammogram of **poly-[TPA-(BTZ)_3_-TPA]** measured in a monomer-free electrolyte at room temperature at a scan rate of 50 mV/s; (**C**) linear dependence of the peak currents on the scan rate.

Electrochemical properties of the D-A-A-A-D azomethine triad **TPA-(BTZ)_3_-TPA** were investigated via cyclic voltammetry in a three-electrode cell configuration using a platinum working electrode, an Ag/Ag^+^ reference electrode, and a platinum wire as a counter electrode. The azomethine triad **TPA-(BTZ)_3_-TPA** exhibited one oxidation/reduction wave at a half-wave potential of +0.67 V vs. Fc/Fc^+^ (E_pa_ = +0.7 V, E_pc_ = +0.64 V). It is known that triphenylamine-based compounds undergo oxidative electropolymerization to form electroactive polymers containing tetraphenylbenzidine (TPB) moieties [60,61,62,63,64,65,66,67], and due to this, the azomethine triad **TPA-(BTZ)_3_-TPA** has been subjected to multiple oxidation/reduction cycles to investigate its ability to electropolymerization (Figure 4A). The concentration of the monomer solution was 1.2 mmol/dm^3^ (1.5 mg/mL), and the electropolymerization was carried out via potentiodynamic cycling at a scan rate of 100 mV/s. Ten oxidation/reduction cycles were conducted, and this process resulted in the formation of orange film on the electrode surface. The electropolymerization of azomethine **TPA-(BTZ)_3_-TPA** and formation of the polymer **poly-[TPA-(BTZ)_3_-TPA]** on the electrode surface have been confirmed by the appearance of a new, reversible oxidation/reduction wave at lower potentials compared to a monomer and an increase in the current of the oxidation/reduction peaks. This resulted in the deposition of the orange film onto the surface of the Pt working electrode. 

After electrochemical polymerization, in order to investigate the electrochemical behavior of **poly-[TPA-(BTZ)_3_-TPA]**, it was subjected to cyclic voltammetry in a monomer-free electrolyte (Figure 4B). During a CV scan in the anodic regime, the polymer exhibited two oxidation/reduction waves at E_1/2_ = +0.49 V and +0.68 V assigned to the stepwise oxidation of TPB units to radical cation and dication, respectively [68,69]. The peak-to-peak separation (ΔE) was found to be 0.053 V and 0.037 V for the first and second oxidation processes, respectively, indicating a fully reversible character of the electrochemical process. 

Further characterization of the redox processes of **poly-[TPA-(BTZ)_3_-TPA]** was conducted by recording the cyclic voltammograms at twelve different scan rates (Figure S14). A linear correlation with the linear correlation coefficients (R^2^) of 0.997 for E_pa_ and 0.980 for E_pc_ between current density measured for the second oxidation/reduction wave and scan rate was observed for **poly-[TPA-(BTZ)_3_-TPA]** in a monomer-free electrolyte solution (Figure 4C). This proves that the polymer layer was well adhered onto the electrode, and redox processes are confined to the electrode surface [70,71,72,73]. 

**Figure 5:**
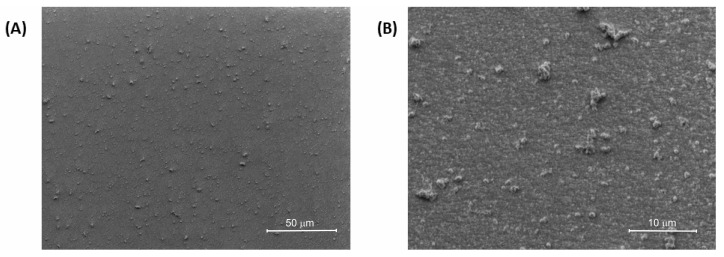
SEM images of the thin film of polyazomethine **poly-[TPA-(BTZ)_3_-TPA]** with magnification (**A**) 1500× and (**B**) 10,000×.

Next, the layer of polyazomethine **poly-[TPA-(BTZ)_3_-TPA]** was deposited on the ITO electrode, and the morphology of the polymer film was investigated using scanning electron microscopy (SEM). The electrodeposition of the polymer on an ITO electrode was conducted at the same conditions as electropolymerization on the Pt electrode (the concentration of the monomer solution was 1.2 mmol/dm^3^, the scan rate was 100 mV/s, and 10 oxidation/reduction cycles were conducted to obtain a polymer layer). The images are shown in Figure 5. The surface of the polymer was found to be smooth and homogenous without any cracks, and it is similar to morphologies observed for another polymers obtained via electropolymerization of triphenylamine-based monomers [64,74,75,76]. 

The average film thickness was investigated using the AFM scratch method. For this, the film was cut with a blade and the difference between bare ITO and the polymer surface in four different places was measured using AFM (Figure S15). The measured average film thickness was found to be ~125 nm.

### 2.4. Spectroelectrochemical Properties

**Figure 6:**
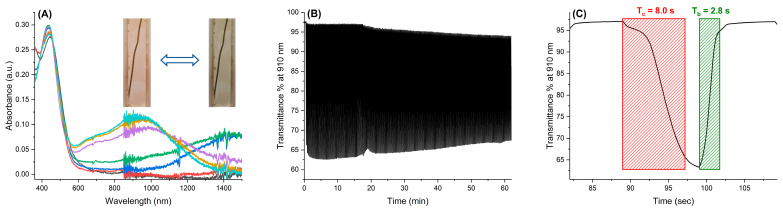
(**A**) Spectroelectrochemistry of **poly-[TPA-(BTZ)_3_-TPA]** measured in an anhydrous and deaerated 0.1 M solution of TBAPF_6_ in dichloromethane as the supporting electrolyte with applied potentials of 0 (**—**), +0.4 (**—**), +0.5 (**—**); +0.6 (**—**), +0.7 (**—**), +0.8 (**—**), and +0.9 (**—**) V vs. Fc/Fc^+^. Insert: photograph showing the **poly-[TPA-(BTZ)_3_-TPA]** in its neutral (left) and oxidized, by applying voltage of +0.8V, (right) states; (**B**) electrochromic stability of the **poly-[TPA-(BTZ)_3_-TPA]** by switching between +0.8 V and −0.1 V in 10 s intervals, monitored at 910 nm; (**C**) coloration and bleaching times of **poly-[TPA-(BTZ)_3_-TPA]**.

To further analyze the redox processes of **poly-[TPA-(BTZ)_3_-TPA]**, spectroelectrochemical characterization was applied. For this, the polymer was deposited on ITO-coated glass slides, and modified this way, the ITO electrode was used as a working electrode for spectroelectrochemical measurements. The electropolymerization conditions were the same as described before. The neutral film was oxidized in a stepwise manner, and UV-Vis-NIR absorption spectra were recorded between 0 V and +1.0 V in a monomer-free electrolyte solution. The UV-Vis-NIR spectra of **poly-[TPA-(BTZ)_3_-TPA]** in different redox states are shown in Figure 6A. 

The neutral form of the polymer was characterized by one absorption band in the visible range at 444 nm. This absorption band is attributed to the intramolecular charge transfer (ICT) between the donors and acceptors. During stepwise oxidation, the broad absorption band in the NIR region (~1460 nm) arises, while the signal connected with neutral form of **poly-[TPA-(BTZ)_3_-TPA]** remains almost unchanged. This is the result of the formation of the radical cation on the TPB group, which is characterized by the presence of intervalence charge transfer (IV-CT) absorption in the NIR region. Because the optical changes occur only at the NIR region, no visible color change was observed during this electrochemical process. Further increase in applied potential results in the decrease of the IV-CT band and formation of a broad absorption band spreading over visible and NIR regions with a maximum at 910 nm. During this process the formation of the TPB dication occurs, and it was accompanied with the color change from orange to green. The color change was fully reversible, and the application of slightly negative potential (−0.1 V) resulted in the color change from green to orange. 

To further investigate the electrochromic parameters of the electropolymerized film, the potential was switched multiple times between its oxidized (+0.8 V) and reduced (−0.1 V) states, and the transmittance of the polymer film was monitored at 910 nm. This allowed for the investigation of the electrochromic stability of the polymer layer, as well as the transmittance difference, switching times, and coloration efficiency. **Poly-[TPA-(BTZ)_3_-TPA]** showed an optical contrast of 35% at 910 nm, and the optical contrast decreased to 27% after ~180 oxidation/reduction cycles, indicating good long-term electrochromic stability (Figure 6B). The response times, T_c90_ and T_b90_, were calculated as the times required to reach 90% of the final change in transmittance difference, and they were found to be 8.0 s and 2.8 s for the coloration and bleaching process, respectively (Figure 6C). These switching times are comparable to those observed for other triphenylamine-based polyazomethines [13,77,78,79]. The coloration efficiency, which is another important parameter characterizing electrochromic materials, has been calculated as a quotient of the optical density (∆OD) of the polymer and charge extracted during the redox process ($Q_{d}$) per unit area, according to the following equation:

$$CE=\frac{∆OD}{Q_{d}}$$

The optical density has been calculated as the logarithm of the ratio of transmittance of the polymer in its bleached ($T_{b}$) and colored ($T_{c}$) states:

$$∆OD=log(\frac{T_{b}}{T_{c}})$$

It was found that the coloration efficiency of **poly-[TPA-(BTZ)_3_-TPA]** is 146 cm^2^/C, which is a comparable value to other polyazomethines [13,77,78]. 

## 3. Materials and Methods

The chemical reagents were purchased from Merck or Fluorochem without additional purification. Tetrahydrofuran (THF), utilized for the synthesis of compound **C**, was purified by refluxing with sodium and benzophenone until the solvent became anhydrous, indicated by a color change to deep blue-purple. The solvent was subsequently distilled under an argon atmosphere and stored in a Schlenk flask. Nuclear magnetic resonance (NMR) spectra were acquired using Bruker Advance 600 MHz and Bruker UltraShield 300 MHz spectrometers. Mass spectrometry analysis was conducted on a QTOF mass spectrometer (Impact HD Bruker, Bruker Daltonics, Bremen, Germany). Absorption and fluorescence measurements were performed using a UV-Vis-NIR Jasco V-770 spectrometer and a Jasco FP-8500 spectrofluorometer, respectively. Fluorescence quantum yields were determined via an absolute method employing a 100 mm diameter integrating sphere, calibrated with a standard halogen–tungsten light source. Electrochemical measurements were conducted with a BioLogic VSP electrochemical workstation. The tested compound was dissolved in anhydrous and deaerated dichloromethane at a concentration of 1 mM along with TBAPF_6_ (0.1 M). Dichloromethane for electrochemistry was purified by refluxing under an argon atmosphere over calcium hydride followed by distillation [80]. The solvent was stored in a Schlenk flask over 4 Å molecular sieves. A platinum electrode was used as the working electrode while platinum wire and a saturated Ag/AgCl electrode were employed as auxiliary and reference electrodes, respectively. The reference electrode was calibrated using ferrocene, and the cyclic voltammograms were recalculated vs Fc/Fc^+^ redox couple. ITO glass slides for on-substrate polymerization studies were obtained from Delta Technologies Ltd. (Loveland, CO, USA), and they were cleaned before use.

*4-(4-Bromo-2,1,3-benzothiadiazol-7-yl)-N,N-diphenylaniline* **A**:

In a two-neck round-bottom flask, 244.5 mg (0.8 mmol) of 4-(diphenylamino)phenylboronic acid, 248.6 mg (0.8 mmol) of 4,7-dibromo-2,1,3-benzothiadiazole, and 470 mg (3.4 mmol) of potassium carbonate were placed. The system was degassed using a three-cycle vacuum/argon procedure, and then the solvent—a mixture of toluene and water in a 2:1 volume ratio—was added via a syringe. The solution was degassed with a stream of argon for 15 min, and the catalyst tetrakis(triphenylphosphine)palladium(0) (48.8 mg, 5 mol%) was added. The mixture was heated at 90 °C for 24 h. After cooling to room temperature, dichloromethane (30 mL) was added, and the mixture was extracted with water (3 × 20 mL). The organic layer was dried over anhydrous magnesium sulfate, filtered, and the solvent was evaporated under reduced pressure using a rotary evaporator. Compound **A** was purified on a chromatography column filled with silica gel. The separation was performed using a mixture of hexane and dichloromethane in a 3:1 volume ratio as the eluent. A total of 275 mg of compound **A** was obtained as a red powder (yield 71%). ^1^H NMR (600 MHz, CDCl_3_) δ 7.90 (d, *J* = 7.6 Hz, 1H), 7.80 (d, *J* = 8.8 Hz, 2H), 7.55 (d, *J* = 7.6 Hz, 1H), 7.30 (dd, *J* = 8.5, 7.4 Hz, 4H), 7.20–7.17 (m, 6H), 7.08 (tt, *J* = 7.3, 1.2 Hz, 2H) ppm. ^13^C NMR (150 MHz, CDCl_3_) δ 154.1, 153.3, 148.6, 147.5, 133.7, 132.5, 130.0, 130.0; 129.5, 127.5; 125.2, 123.7, 122.8, 112.3 ppm. HRMS m/z = calculated 457.0243; found 457.0217 (M)^+^. Elemental analysis calculated for C_24_H_16_BrN_3_S (457.0248): calculated: C, 62.89; H, 3.52; N, 9.17; S, 7.00; found: C, 62.88, H, 3.54; N, 9.18; S, 7.02%.

*4-(4-Phenylamino-2,1,3-benzothiadiazol-7-yl)-N,N-diphenylaniline* **B**:

In a two-neck round-bottom flask, 275 mg of compound **A** (0.6 mmol), 198 mg (0.9 mmol) of 4-aminophenylboronic acid pinacol ester, and 331.2 mg of potassium carbonate (2.4 mmol) were placed. The system was degassed using a three-cycle vacuum/argon procedure, and the solvent (toluene/water in a 2:1 volume ratio) was added via syringe. The mixture was degassed with a stream of argon for 15 min, followed by the addition of 22.5 mg of the catalyst tetrakis(triphenylphosphine)palladium(0) (5 mol%) and 10 mg of the phase transfer catalyst tetrabutylammonium bromide. The mixture was heated at 90 °C for 24 h. After cooling to room temperature, dichloromethane (30 mL) was added to the mixture, and it was extracted with water (3 × 20 mL). The organic layer was dried over anhydrous magnesium sulfate, filtered, and the solvent was evaporated under reduced pressure using a rotary evaporator. Compound **B** was purified on a chromatography column filled with silica gel. The separation was performed using a dichloromethane/hexane mixture in a 2:1 volume ratio as the eluent. After isolating the unreacted substrate **A**, the polarity of the eluent was increased, and a dichloromethane/hexane mixture in a 3:1 volume ratio was used. A total of 165 mg of compound **B** was obtained as a yellow powder (yield 60%). ^1^H NMR (600 MHz, CDCl_3_) δ 7.87 (d, *J* = 8.9 Hz, 2H), 7.83 (d, *J* = 8.7 Hz, 2H), 7.72 (d, *J* = 7.4 Hz, 1H), 7.70 (d, *J* = 7.4 Hz, 1H), 7.29 (dd, *J* = 8.6, 7.3 Hz, 4H), 7.24–7.17 (m, 6H), 7.09–7.04 (m, 2H), 6.85 (d, *J* = 8.7 Hz, 2H), 3.85 (s, 2H) ppm. ^13^C NMR (151 MHz, CDCl_3_) δ 154.5, 154.3, 148.0, 147.7, 146.9, 132.9, 131.8, 131.4, 130.4, 130.0, 129.5, 127.9, 127.7, 126.9, 124.0, 123.4, 123.2, 115.2 ppm. HRMS *m*/*z* = calculated 471.1638; found 471.1636 (M+H)^+^. Elemental analysis calculated for C_30_H_22_N_4_S (470.1565): calculated: C, 76.57; H, 4.71; N, 11.91; S, 6.81; found: C, 76.56; H, 4.73; N, 11.90; S, 6.82%.

*4-[4-(4-formylphenyl)-2,1,3-benzothiadiazol-7-yl]benzaldehyde* **C**:

The benzothiadiazole-based dialdehyde **C** has been prepared in similar way as described in the literature [81,82,83,84]. In a two-neck round-bottom flask, 200 mg (0.68 mmol) of 4,7-dibromo-2,1,3-benzothiadiazole, 255 mg (1.7 mmol) of 4-formylbenzeneboronic, and 370 mg of sodium carbonate (3.5 mmol) were placed. The system was degassed using a three-cycle vacuum/argon procedure, and the solvent (toluene/water in a 2:1 volume ratio) was added via syringe. The mixture was degassed with a stream of argon for 15 min, followed by the addition of 80 mg of the catalyst tetrakis(triphenylphosphine)palladium(0) (10 mol%) and 10 mg of the phase transfer catalyst tetrabutylammonium bromide. The mixture was heated at 95 °C for 24 h. After cooling to room temperature, dichloromethane (35 mL) was added to the mixture, and it was extracted with water (3 × 25 mL). The organic layer was dried over anhydrous magnesium sulfate, filtered, and the solvent was evaporated under reduced pressure using a rotary evaporator. Compound **C** was purified on a chromatography column filled with silica gel. The separation was performed using a dichloromethane/hexane mixture in a 1:1 volume ratio as the eluent. A total of 160 mg of compound **C** was obtained as a yellow powder (yield 68%). ^1^H NMR (300 MHz, DMSO) δ 10.12 (s, 2H), 8.28 (d, *J* = 8.3 Hz, 4H), 8.13 (d, *J* = 4.6 Hz, 4H), 8.09 (s, 2H) ppm. ^13^C NMR (75 MHz, DMSO) δ 192.86, 153.13, 142.34, 135.76, 131.98, 129.92, 129.21 ppm. HRMS *m/z* = calcd 343.0546; found 343.0299 (M)^−^. Elemental analysis calculated for C_20_H_12_N_2_O_2_S (344.0619): calculated: C, 69.75; H, 3.51; N, 8.13; S, 9.31; found: C, 69.77, H, 3.55; N, 8.12; S, 9.30%.

*Azomethine triad* **TPA-(BTZ)_3_-TPA**:

In a two-neck round-bottom flask, 11.9 mg (0.03 mmol) of 4-[4-(4-formylphenyl)-2,1,3-benzothiadiazol-7-yl]benzaldehyde **C** and 38.7 mg (0.07 mmol) of compound **B** were placed. The flask was set on a magnetic stirrer, and the system was degassed using a three-cycle vacuum/argon procedure. Then, anhydrous and deoxygenated THF (5 mL) and 1.74 mg of scandium(III) trifluoromethanesulfonate (5 mol%) were added. The reaction was carried out at room temperature for 24 h. Upon completion, an orange precipitate formed, which was centrifuged and washed with hexane. This resulted in 25 mg of compound **C** as an orange precipitate (yield 58%). ^1^H NMR (600 MHz, CDCl_3_) δ 8.67 (s, 2H), 8.16 (d, *J* = 4.9 Hz, 4H), 8.09–8.06 (m, 4H), 7.93 (s, 2H), 7.90 (d, *J* = 8.8 Hz, 4H), 7.84 (d, *J* = 7.2 Hz, 2H), 7.79 (d, *J* = 7.4 Hz, 2H), 7.46 (d, *J* = 8.4 Hz, 4H), 7.32–7.29 (m, 8H), 7.24–7.18 (m, 16H), 7.09–7.06 (m, 4H). ^13^C NMR (151 MHz, CDCl_3_) δ 159.9; 154.2; 154.1; 151.9; 148.1; 147.5; 140.2; 137.9; 136.2; 133.0; 132.8; 132.1; 130.1; 129.9; 129.7; 129.4; 129.3; 129.2; 128.4; 128.0; 127.3; 124.9; 124.8; 123.3; 122.9; 121.3 ppm. HRMS *m*/*z* = calculated 1249.3617; found 1249.3605 (M+H)^+^. Elemental analysis calculated for C_80_H_52_N_10_S_3_ (1248.354) calculated: C, 76.90; H, 4.19; N, 11.21; S, 7.70; found: C, 76.92; H, 4.25; N, 11.19; S, 7.69%.

## 4. Conclusions

In summary, the synthesis, electropolymerization, and optoelectrical properties of a D-A-A-A-D azomethine **TPA-(BTZ)_3_-TPA** were presented. The azomethine was found to exhibit luminescence properties with high quantum yields and a positive solvatochromic effect. Azomethine also exhibited emission in the solid state with the maxima in an orange region of the electromagnetic spectrum. The multiple oxidation/reduction cycles of azomethine resulted in formation of thin layer of polymer **poly-[TPA-(BTZ)_3_-TPA]** on the electrode surface. This was due to the coupling of the terminal triphenylamine groups and formation of tetraphenylbenzidine moieties. The polymer **poly-[TPA-(BTZ)_3_-TPA]** exhibited reversible oxidation/reduction typical to polymers containing bis(triphenylamine) groups. The electrochromism from orange to green and from no to strong near-infrared absorption are achieved from this azomethine polymer film. The polyazomethine polymer **poly-[TPA-(BTZ)_3_-TPA]** exhibited an optical contrast of 35% at 910 nm and good electrochromic stability over ~180 oxidation/reduction cycles. The response times and coloration efficiency were found to be comparable to other polyazomethines. Visible-NIR activity of the polymer makes it an ideal candidate for application, for example, in smart windows [85], which could regulate the intensity of the transmitted NIR radiation leading to the reduction in air conditioning costs and providing environmental benefits [86]. Observed response time are sufficient for application in smart windows, whose acceptable switching times are on the order of minutes [87]. 

## Figures and Tables

**Table 1:** The optical properties of the azomethine triad **TPA-(BTZ)_3_-TPA**.

Solvent	λ_abs_ (nm)	λ_em_ (nm)	Stokes Shift (cm^−1^)	Quantum Yield (%)
Toluene	443	577	5242	35
Dioxane	438	580	5590	37
Tetrahydrofuran	439	602	6168	32
DCM	435	618	6808	27

## Data Availability

Data are contained within this article or Supplementary Material.

## Supplementary Materials

The following supporting information can be downloaded at: https://www.mdpi.com/article/10.3390/molecules29184470/s1, Figure S1: ^1^H NMR of TPA-(BTZ)_3_-TPA azomethine in CDCl_3_; Figure S2: ^13^C NMR of TPA-(BTZ)_3_-TPA azomethine in CDCl_3_; Figure S3: HR-MS of TPA-(BTZ)_3_-TPA azomethine; Figure S4: ^1^H NMR of A in CDCl_3_; Figure S5: ^13^C NMR of A in CDCl_3_; Figure S6: HR-MS of A; Figure S7: ^1^H NMR of B in CDCl_3_; Figure S8: ^13^C NMR of B in CDCl_3_; Figure S9: HR-MS of B; Figure S10: ^1^H NMR of C in d_6_-DMSO; Figure S11: ^13^C NMR of C in d_6_-DMSO; Figure S12: HR-MS spectra of C; Figure S13: Absorption spectra of azomethine in toluene (black), dioxane (red), THF (blue), and dichloromethane (green); Figure S14: CV profiles of polyazomethine obtained at different scan rates; Figure S15: (A) AFM micrograph of **poly-[TPA-(BTZ)_3_-TPA]** deposited on ITO electrode showing the step between the ITO and the polymer surface. AFM cross-section profiles were measured at marked places; (B) AFM profiles measured at places shown in (A).

## References

[1] Bolduc A., Mallet C., Skene W.G. (2013). Survey of recent advances of in the field of π-conjugated heterocyclic azomethines as materials with tuneable properties. Sci. China Chem..

[2] Yang C.J., Jenekhe S.A. (1991). Conjugated aromatic poly(azomethines). 1. Characterization of structure, electronic spectra, and processing of thin films from soluble complexes. Chem. Mater..

[3] Bolduc A., Al Ouahabi A., Mallet C., Skene W.G. (2013). Insight into the Isoelectronic Character of Azomethines and Vinylenes Using Representative Models: A Spectroscopic and Electrochemical Study. J. Org. Chem..

[4] Charland-Martin A., Collier G.S. (2024). Understanding Degradation Dynamics of Azomethine-containing Conjugated Polymers. Macromolecules.

[5] Filiatrault H.L., Muras K., Wałęsa-Chorab M., Skene W.G. (2024). On-Substrate Preparation of a Poly(triphenylamino azomethine) for Electrochromic Devices. Polymers.

[6] Wałęsa-Chorab M., Skene W.G. (2024). Leveraging reversible bonds for property modification of electrochromes and their immobilization by dual modes: Thermal and electrochemical polymerization. Prog. Org. Coatings.

[7] Lerond M., Bélanger D., Skene W.G. (2017). Surface immobilized azomethine for multiple component exchange. Soft Matter.

[8] Wałȩsa-Chorab M., Skene W.G. (2020). Engaging the reversible bonds of an immobilized styreno-thiophene film. Cryst. Growth Des..

[9] Abbasi A., Rezvani Z., Nejati K. (2006). Synthesis and properties of new liquid crystalline compounds containing an alkoxyphenylazo group. Dyes Pigment..

[10] Ng S.C., Chan H.S.O., Wong P.M.L., Tan K.L., Tan B.T.G. (1998). Novel heteroarylene polyazomethines: Their syntheses and characterizations. Polymer.

[11] Fujii S., Minami S., Urayama K., Suenaga Y., Naito H., Miyashita O., Imoto H., Naka K. (2018). Beads-on-String-Shaped Poly(azomethine) Applicable for Solution Processing of Bilayer Devices Using a Same Solvent. ACS Macro Lett..

[12] Suh S.C., Shim S.C. (2000). Synthesis and properties of a novel polyazomethine, the polymer with high photoconductivity and second-order optical nonlinearity. Synth. Met..

[13] Napierała S., Kubicki M., Wałęsa-Chorab M. (2021). Toward Electrochromic Metallopolymers: Synthesis and Properties of Polyazomethines Based on Complexes of Transition-Metal Ions. Inorg. Chem..

[14] Sicard L., Navarathne D., Skalski T., Skene W.G. (2013). On-Substrate Preparation of an Electroactive Conjugated Polyazomethine from Solution-Processable Monomers and its Application in Electrochromic Devices. Adv. Funct. Mater..

[15] Wałęsa-Chorab M., Skene W.G. (2019). Investigation of an electroactive immobilized azomethine for potential electrochromic use. Sol. Energy Mater. Sol. Cells.

[16] Leliège A., Barik S., Skene W.G. (2014). Photopatternable Electrochromic Materials from Oxetane Precursors. ACS Appl. Mater. Interfaces.

[17] Sonker E., Tiwari R., Kumar K., Krishnamoorthi S. (2020). Electrical properties of new polyazomethines. SN Appl. Sci..

[18] Garbay G., Giraud L., Gali S.M., Hadziioannou G., Grau E., Grelier S., Cloutet E., Cramail H., Brochon C. (2020). Divanillin-Based Polyazomethines: Toward Biobased and Metal-Free π-Conjugated Polymers. ACS Omega.

[19] Li G., Yu K., Noordijk J., Meeusen-Wierts M.H.M., Gebben B., oude Lohuis P.A.M., Schotman A.H.M., Bernaerts K.V. (2020). Hydrothermal polymerization towards fully biobased polyazomethines. Chem. Commun..

[20] Wałȩsa-Chorab M., Skene W.G. (2014). On-substrate polymerization-a versatile approach for preparing conjugated polymers suitable as electrochromes and for metal ion sensing. RSC Adv..

[21] Leung A.C.W., MacLachlan M.J. (2007). Schiff Base Complexes in Macromolecules. J. Inorg. Organomet. Polym. Mater..

[22] Ketcham K.A., Swearingen J.K., Castiñeiras A., Garcia I., Bermejo E., West D.X. (2001). Iron(III), cobalt(II,III), copper(II) and zinc(II) complexes of 2-pyridineformamide 3-piperidylthiosemicarbazone. Polyhedron.

[23] Pająk A.K., Kotowicz S., Gnida P., Małecki J.G., Ciemięga A., Łuczak A., Jung J., Schab-Balcerzak E. (2022). Synthesis and Characterization of New Conjugated Azomethines End-Capped with Amino-thiophene-3,4-dicarboxylic Acid Diethyl Ester. Int. J. Mol. Sci..

[24] Iwan A., Palewicz M., Chuchmała A., Gorecki L., Sikora A., Mazurek B., Pasciak G. (2012). Opto(electrical) properties of new aromatic polyazomethines with fluorene moieties in the main chain for polymeric photovoltaic devices. Synth. Met..

[25] Hindson J.C., Ulgut B., Friend R.H., Greenham N.C., Norder B., Kotlewski A., Dingemans T.J. (2010). All-aromatic liquid crystal triphenylamine-based poly(azomethine)s as hole transport materials for opto-electronic applications. J. Mater. Chem..

[26] Petrus M.L., Bouwer R.K.M., Lafont U., Athanasopoulos S., Greenham N.C., Dingemans T.J. (2014). Small-molecule azomethines: Organic photovoltaics via Schiff base condensation chemistry. J. Mater. Chem. A.

[27] Dufresne S., Skene W.G. (2012). Optoelectronic property tailoring of conjugated heterocyclic azomethines—The effect of pyrrole, thiophene and furans. J. Phys. Org. Chem..

[28] Schab-Balcerzak E., Iwan A., Krompiec M., Siwy M., Tapa D., Sikora A., Palewicz M. (2010). New thermotropic azomethine–naphthalene diimides for optoelectronic applications. Synth. Met..

[29] Amin M.F., Gnida P., Kotowicz S., Małecki J.G., Siwy M., Nitschke P., Schab-Balcerzak E. (2022). Spectroscopic and Physicochemical Investigations of Azomethines with Triphenylamine Core towards Optoelectronics. Materials.

[30] Chen C.-K., Lin Y.-C., Ho J.-C., Yang W.-C., Chen W.-C. (2022). Biomass-Derived Degradable Poly(azomethine)s for Flexible Bistable Photonic Transistor Memories. ACS Sustain. Chem. Eng..

[31] Pan L., Hu B., Zhu X., Chen X., Shang J., Tan H., Xue W., Zhu Y., Liu G., Li R.-W. (2013). Role of oxadiazole moiety in different D–A polyazothines and related resistive switching properties. J. Mater. Chem. C.

[32] Liu C.-L., Chen W.-C. (2015). Conjugated Polymers for Memory Device Applications. Electrical Memory Materials and Devices.

[33] Cai G., Wang J., Lee P.S. (2016). Next-Generation Multifunctional Electrochromic Devices. Acc. Chem. Res..

[34] Yıldırım M., Kaya İ., Aydın A. (2013). Azomethine coupled fluorene–thiophene–pyrrole based copolymers: Electrochromic applications. React. Funct. Polym..

[35] Gautier Y., Skene W.G. (2024). Effect of azomethine structural modification of electrochromic performance. J. Mater. Chem. C.

[36] Koole M., Frisenda R., Petrus M.L., Perrin M.L., van der Zant H.S.J., Dingemans T.J. (2016). Charge transport through conjugated azomethine-based single molecules for optoelectronic applications. Org. Electron..

[37] Ma X., Niu H., Wen H., Wang S., Lian Y., Jiang X., Wang C., Bai X., Wang W. (2015). Synthesis, electrochromic, halochromic and electro-optical properties of polyazomethines with a carbazole core and triarylamine units serving as functional groups. J. Mater. Chem. C.

[38] Farcas A., Jarroux N., Ghosh I., Guégan P., Nau W.M., Harabagiu V. (2009). Polyrotaxanes of Pyrene–Triazole Conjugated Azomethine and α-Cyclodextrin with High Fluorescence Properties. Macromol. Chem. Phys..

[39] Georgiev A., Yordanov D., Dimov D., Zhivkov I., Nazarova D., Weiter M. (2020). Azomethine phthalimides fluorescent E→Z photoswitches. J. Photochem. Photobiol. A Chem..

[40] Bishop S., Tremblay M.-H., Gellé A., Skene W.G. (2019). Understanding Color Tuning and Reversible Oxidation of Conjugated Azomethines. J. Phys. Chem. A.

[41] Marin L., van der Lee A., Shova S., Arvinte A., Barboiu M. (2015). Molecular amorphous glasses toward large azomethine crystals with aggregation-induced emission. New J. Chem..

[42] Krishnaveni K., Gurusamy S., Rajakumar K., Sathish V., Thanasekaran P., Mathavan A. (2022). Aggregation induced emission (AIE), selective fluoride ion sensing and lysozyme interaction properties of Julolidinesulphonyl derived Schiff base. J. Photochem. Photobiol. A Chem..

[43] Wałęsa-Chorab M., Tremblay M.H., Skene W.G. (2016). Hydrogen-Bond and Supramolecular-Contact Mediated Fluorescence Enhancement of Electrochromic Azomethines. Chemistry.

[44] Gather M.C., Köhnen A., Meerholz K. (2011). White Organic Light-Emitting Diodes. Adv. Mater..

[45] Dufresne S., Callaghan L., Skene W.G. (2009). Conjugated Fluorenes Prepared from Azomethines Connections-II: The Effect of Alternating Fluorenones and Fluorenes on the Spectroscopic and Electrochemical Properties. J. Phys. Chem. B.

[46] Orlova N., Nikolajeva I., Pučkins A., Belyakov S., Kirilova E. (2021). Heterocyclic Schiff Bases of 3-Aminobenzanthrone and Their Reduced Analogues: Synthesis, Properties and Spectroscopy. Molecules.

[47] Tiwari K., Mishra M., Singh V.P. (2014). 8(E)-4-[{2-(2,4-dinitrophenyl)hydrazono}benzene-1,3-diol] as a solvatochromic Schiff base and chromogenic signaling of water content by its deprotonated form in acetonitrile. RSC Adv..

[48] Takeda Y. (2024). Modulating the Photophysical Properties of Twisted Donor–Acceptor–Donor π-Conjugated Molecules: Effect of Heteroatoms, Molecular Conformation, and Molecular Topology. Acc. Chem. Res..

[49] Muras K., Kubicki M., Wałęsa-Chorab M. (2023). Benzochalcodiazole-based donor-acceptor-donor non-symmetric small molecules as dual-functioning electrochromic and electrofluorochromic materials. Dyes Pigment..

[50] Deng B., Guo F., Duan N., Yang S., Tian H., Sun B. (2022). A Solvatochromic Fluorescent Probe for Solvent Polarity Detection Using a Smartphone. ChemistrySelect.

[51] Nath S., Bhattacharya B., Sarkar U., Singh T.S. (2022). Solvent Effects on the Photophysical Properties of a Donor–acceptor Based Schiff Base. J. Fluoresc..

[52] Nath S., Bhattacharya B., Sarkar U., Singh T.S. (2022). Photophysical investigation of a donor-acceptor based Schiff base in solvents of varying polarities. J. Mol. Struct..

[53] Gautam P., Misra R., Koukaras E.N., Sharma A., Sharma G.D. (2015). Donor–acceptor–acceptor–donor small molecules for solution processed bulk heterojunction solar cells. Org. Electron..

[54] Chua M.H., Zhu Q., Tang T., Shah K.W., Xu J. (2019). Diversity of electron acceptor groups in donor–acceptor type electrochromic conjugated polymers. Sol. Energy Mater. Sol. Cells.

[55] Dou L., Liu Y., Hong Z., Li G., Yang Y. (2015). Low-Bandgap Near-IR Conjugated Polymers/Molecules for Organic Electronics. Chem. Rev..

[56] Pluczyk-Malek S., Honisz D., Akkuratov A., Troshin P., Lapkowski M. (2021). Tuning the electrochemical and optical properties of donor-acceptor D-A2-A1-A2-D derivatives with central benzothiadiazole core by changing the A2 strength. Electrochim. Acta.

[57] Kurowska A., Zassowski P., Kostyuchenko A.S., Zheleznova T.Y., Andryukhova K.V., Fisyuk A.S., Pron A., Domagala W. (2017). Effect of donor to acceptor ratio on electrochemical and spectroscopic properties of oligoalkylthiophene 1,3,4-oxadiazole derivatives. Phys. Chem. Chem. Phys..

[58] Chang Z.-F., Jing L.-M., Chen B., Zhang M., Cai X., Liu J.-J., Ye Y.-C., Lou X., Zhao Z., Liu B. (2016). Rational design of asymmetric red fluorescent probes for live cell imaging with high AIE effects and large two-photon absorption cross sections using tunable terminal groups. Chem. Sci..

[59] Barik S., Skene W.G. (2013). Turning-on the Quenched Fluorescence of Azomethines through Structural Modifications. Eur. J. Org. Chem..

[60] Li W., Yuan F., Xu N., Mei S., Chen Z., Zhang C. (2021). Triphenylamine-triazine polymer materials obtained by electrochemical polymerization: Electrochemistry stability, anions trapping behavior and electrochromic-supercapacitor application. Electrochim. Acta.

[61] Nowacki M., Wałęsa-Chorab M. (2023). Influence of temperature on electrochemical and electrochromic properties of naphthalenediimide-triphenylamine-based polymer. Prog. Org. Coat..

[62] Fu W., Chen H., Han Y., Wang W., Zhang R., Liu J. (2021). Electropolymerization of D-A-D type monomers consisting of triphenylamine and substituted quinoxaline moieties for electrochromic devices. New J. Chem..

[63] Fu W., Chen H., Yi X., Zhang R., Liu J. (2022). Electrochemical polymerization of D-A-D type monomers consisting of triphenylamine and benzo[1,2-b:4,5-b′]dipyrazine units for multicolor electrochromism. Eur. Polym. J..

[64] Hsiao S.H., Liao W.K., Liou G.S. (2018). A comparative study of redox-active, ambipolar electrochromic triphenylamine-based polyimides prepared by electrochemical polymerization and conventional polycondensation methods. Polym. Chem..

[65] Chandra Santra D., Mondal S., Malik S. (2016). Design of triphenylamine appended anthracene derivatives: Electro-polymerization and their electro-chromic behaviour. RSC Adv..

[66] Hsiao S.-H., Chen Y.-Z. (2018). Electrosynthesis of redox-active and electrochromic polymer films from triphenylamine-cored star-shaped molecules end-capped with arylamine groups. Eur. Polym. J..

[67] Santra D.C., Nad S., Malik S. (2018). Electrochemical polymerization of triphenylamine end-capped dendron: Electrochromic and electrofluorochromic switching behaviors. J. Electroanal. Chem..

[68] Guven N., Şener Cemaloğlu Ö., Camurlu P. (2022). Fast Switching Triphenylamine-Based Electrochromic Polymers with Fluorene Core: Electrochemical Synthesis and Optoelectronic Properties. J. Electrochem. Soc..

[69] Hsiao S.-H., Lin J.-W. (2014). Facile preparation of electrochromic poly(amine–imide) films from diimide compounds with terminal triphenylamino groups via electrochemical oxidative coupling reactions. Polym. Chem..

[70] Elgrishi N., Rountree K.J., McCarthy B.D., Rountree E.S., Eisenhart T.T., Dempsey J.L. (2018). A Practical Beginner’s Guide to Cyclic Voltammetry. J. Chem. Educ..

[71] Tang J.H., He Y.Q., Shao J.Y., Gong Z.L., Zhong Y.W. (2016). Multistate Redox Switching and Near-Infrared Electrochromism Based on a Star-Shaped Triruthenium Complex with a Triarylamine Core. Sci. Rep..

[72] Wang C., Wang M., Zhang Y., Zhao J., Fu C. (2016). A new electrochromic copolymer which switched between neutral black and oxidized transmissive. RSC Adv..

[73] Yao C.J., Yao J., Zhong Y.W. (2012). Metallopolymeric films based on a biscyclometalated ruthenium complex bridged by 1,3,6,8-tetra(2-pyridyl)pyrene: Applications in near-infrared electrochromic windows. Inorg. Chem..

[74] Wałęsa-Chorab M., Banasz R., Kubicki M., Patroniak V. (2017). Dipyrromethane functionalized monomers as precursors of electrochromic polymers. Electrochim. Acta.

[75] Zhang H., Yao M., Wei J., Zhang Y., Zhang S., Gao Y., Li J., Lu P., Yang B., Ma Y. (2017). Stable p/n-Dopable Conducting Redox Polymers for High-Voltage Pseudocapacitor Electrode Materials: Structure-Performance Relationship and Detailed Investigation into Charge-Trapping Effect. Adv. Energy Mater..

[76] Mangione M.I., Spanevello R.A., Rumbero A., Heredia D., Marzari G., Fernandez L., Otero L., Fungo F. (2013). Electrogenerated Conductive Polymers from Triphenylamine End-Capped Dendrimers. Macromolecules.

[77] Yen H.J., Liou G.S. (2010). Novel blue and red electrochromic poly(azomethine ether)s based on electroactive triphenylamine moieties. Org. Electron..

[78] Liu F., Bai J., Yu G., Ma F., Hou Y., Niu H. (2020). Synthesis, electrochromic properties and flash memory behaviors of novel D-A-D polyazomethines containing EDOT and thiophene units. Org. Electron..

[79] Liu F., Cong Z., Yu G., Niu H., Hou Y., Wang C., Wang S. (2020). Novel D-A-D conjugated polymers based on tetraphenylethylene monomer for electrochromism. Opt. Mater..

[80] Kadish K.M., Anderson J.E. (1987). Purification of solvents for electroanalysis: Benzonitile. Pure Appl. Chem..

[81] Wang L., Xia Q., Hou M., Yan C., Xu Y., Qu J., Liu R. (2017). A photostable cationic fluorophore for long-term bioimaging. J. Mater. Chem. B.

[82] Tang Y., Huang H., Peng B., Chang Y., Li Y., Zhong C. (2020). A thiadiazole-based covalent triazine framework nanosheet for highly selective and sensitive primary aromatic amine detection among various amines. J. Mater. Chem. A.

[83] Deng D., Zou Y., Chen Z., Liu S., Yang Y., Pu S. (2023). Finely regulated benzothiadiazole derivatives: Aggregation-induced emission (AIE), hypso- or bathochromic mechanofluorochromic behaviors, and multilevel information encryption applications. Dyes Pigment..

[84] Song H., Li T.Y., Pan Y., Han X., Guo Y., Shi L., Song M.-P. (2023). Covalent organic nanocage with aggregation induced emission property and detection for Hg^2+^ as fluorescence sensors. Dyes Pigment..

[85] Niu J., Wang Y., Zou X., Tan Y., Jia C., Weng X., Deng L. (2021). Infrared electrochromic materials, devices and applications. Appl. Mater. Today.

[86] Jelle B.P. (2013). Solar radiation glazing factors for window panes, glass structures and electrochromic windows in buildings—Measurement and calculation. Sol. Energy Mater. Sol. Cells.

[87] Gu C., Jia A.-B., Zhang Y.-M., Zhang S.X.-A. (2022). Emerging Electrochromic Materials and Devices for Future Displays. Chem. Rev..

