# Supplementary material for: Electrochemical and Optical Properties of D-A-A-A-D Azomethine Triad and Its NIR-Active Polymer
Source: Molecules. 2024 Sep 20;29(18):4470. doi: 10.3390/molecules29184470 (PMC11434257; doi:10.3390/molecules29184470)
Supplement: Supplementary file 1 [file molecules-29-04470-s001.zip › molecules-3193811-supplementary.pdf]

Supporting information

Electrochemical and optical properties of D-A-A-A-D azomethine triad and its NIR-active polymer

Mateusz Roszyk and Monika Wałesa-Chorab*

Faculty of Chemistry, Adam Mickiewicz University in Poznań, Uniwersytetu Poznańskiego 8, 61-614 Poznań, Poland

*Correspondence: mchorab@amu.edu.pl

Table of content

Figure S1. ^{1}H NMR of **TPA-(BTZ)$_3$-TPA** azomethine in $CDCl_3$. 3
Figure S2. ^{13}C NMR of **TPA-(BTZ)$_3$-TPA** azomethine in $CDCl_3$. 3
Figure S3. HR-MS of **TPA-(BTZ)$_3$-TPA** azomethine. 4
Figure S4. ^{1}H NMR of **A** in $CDCl_3$. 4
Figure S5. ^{13}C NMR of **A** in $CDCl_3$. 5
Figure S6. HR-MS of **A**. 5
Figure S7. ^{1}H NMR of **B** in $CDCl_3$. 6
Figure S8. ^{13}C NMR of **B** in $CDCl_3$. 6
Figure S9. HR-MS of **B**. 7
Figure S10. ^{1}H NMR of **C** in d_6-DMSO. 7
Figure S11. ^{13}C NMR of **C** in d_6-DMSO. 8
Figure S12. HR-MS spectra of **C**. 8
Figure S13. Absorption spectra of azomethine in toluene (black), dioxane (red), THF (blue) and dichloromethane (green). 9
Figure S14. CV profiles of polyazomethine obtained at different scan rates. 9
Figure S15. A) AFM micrograph of **poly-[TPA-(BTZ)$_3$-TPA]** deposited on ITO electrode showing the step between the ITO and the polymer surface. AFM cross-section profiles were measured at a marked places; B) AFM profiles measured at places shown in A). 10

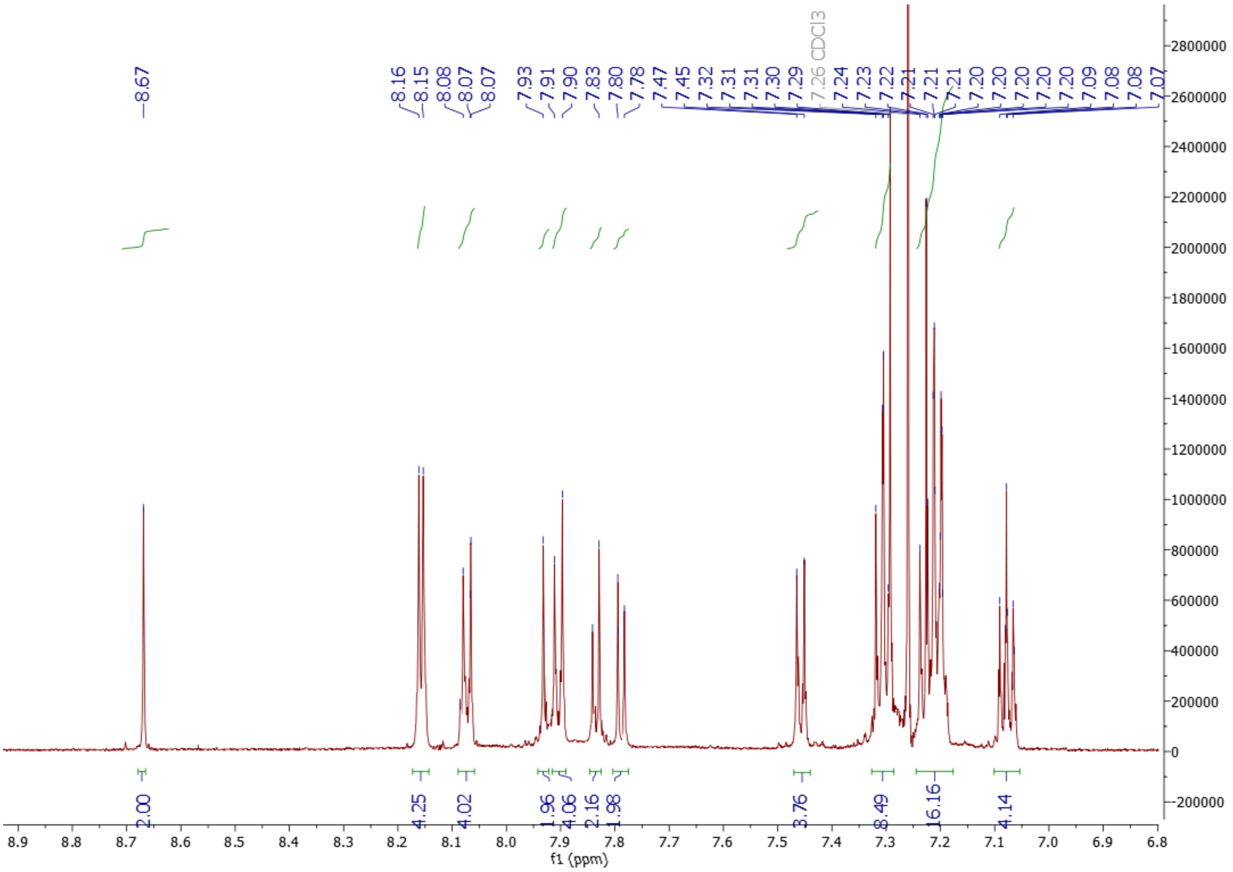

Figure S1. ^{1}H NMR of **TPA-(BTZ)$_3$-TPA** azomethine in $CDCl_3$.

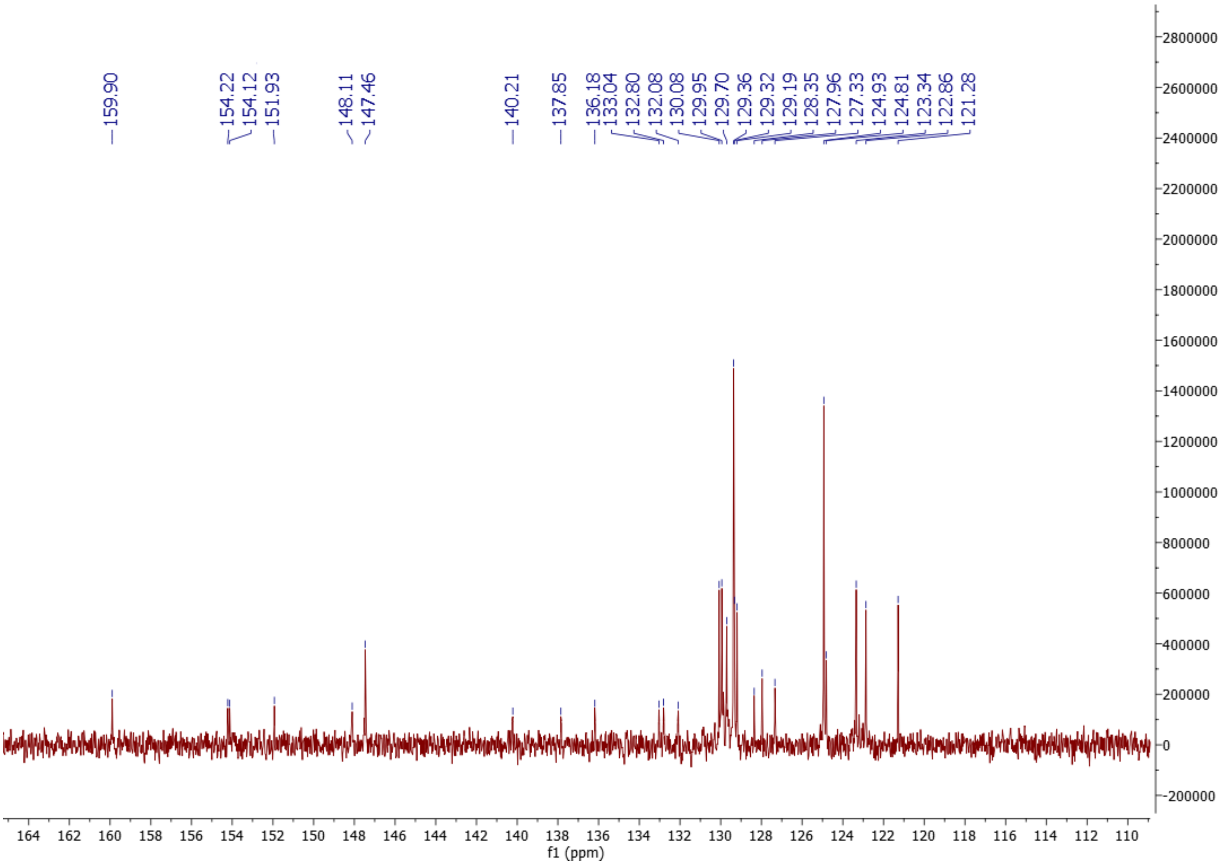

Figure S2. ^{13}C NMR of **TPA-(BTZ)$_3$-TPA** azomethine in $CDCl_3$.

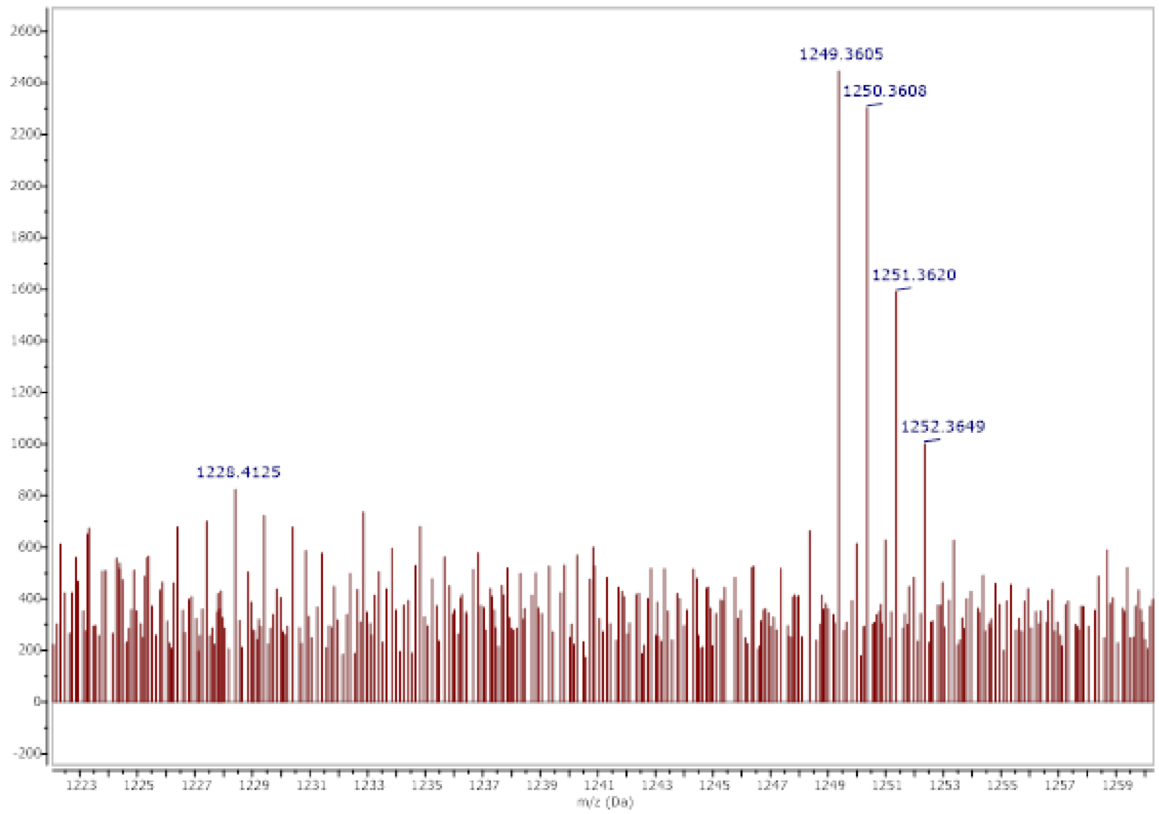

Figure S3. HR-MS of **TPA-(BTZ)₃-TPA** azomethine.

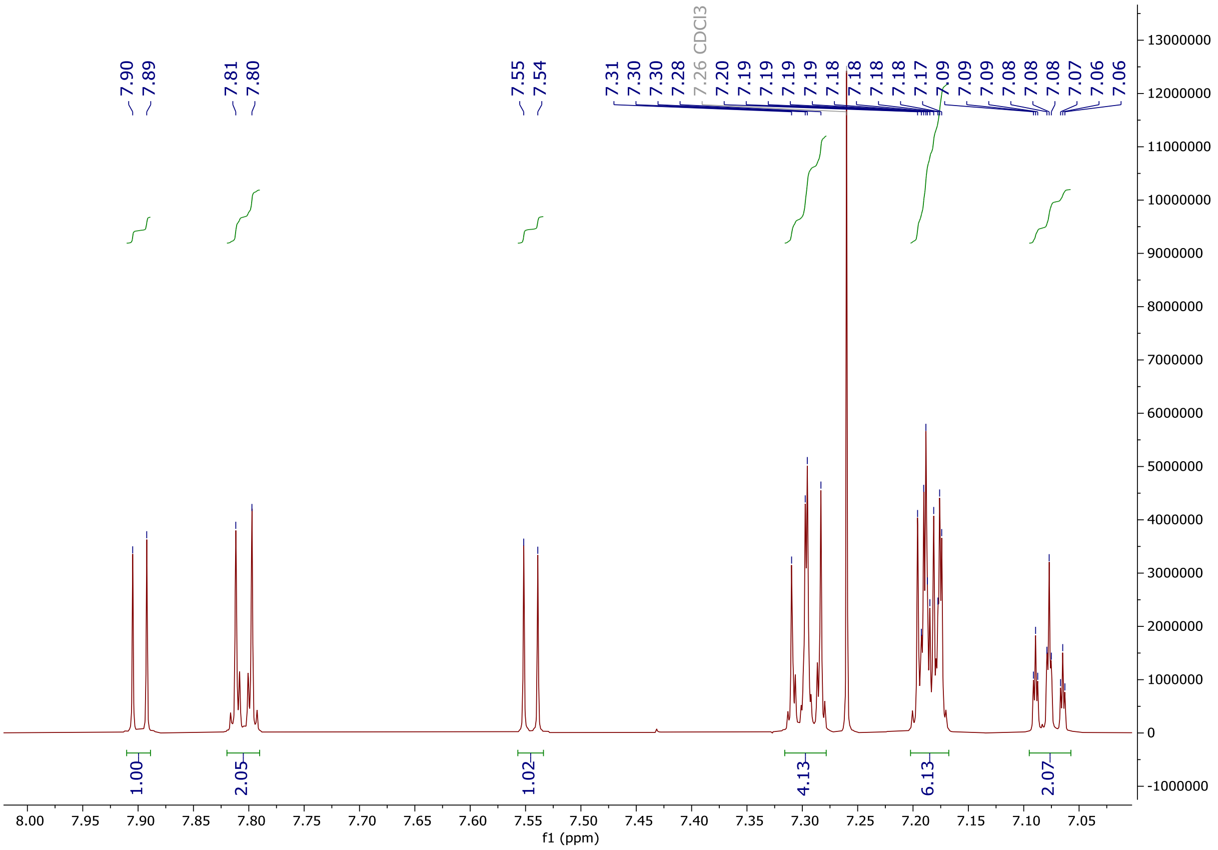

Figure S4. ^{1}H NMR of **A** in $CDCl_3$.

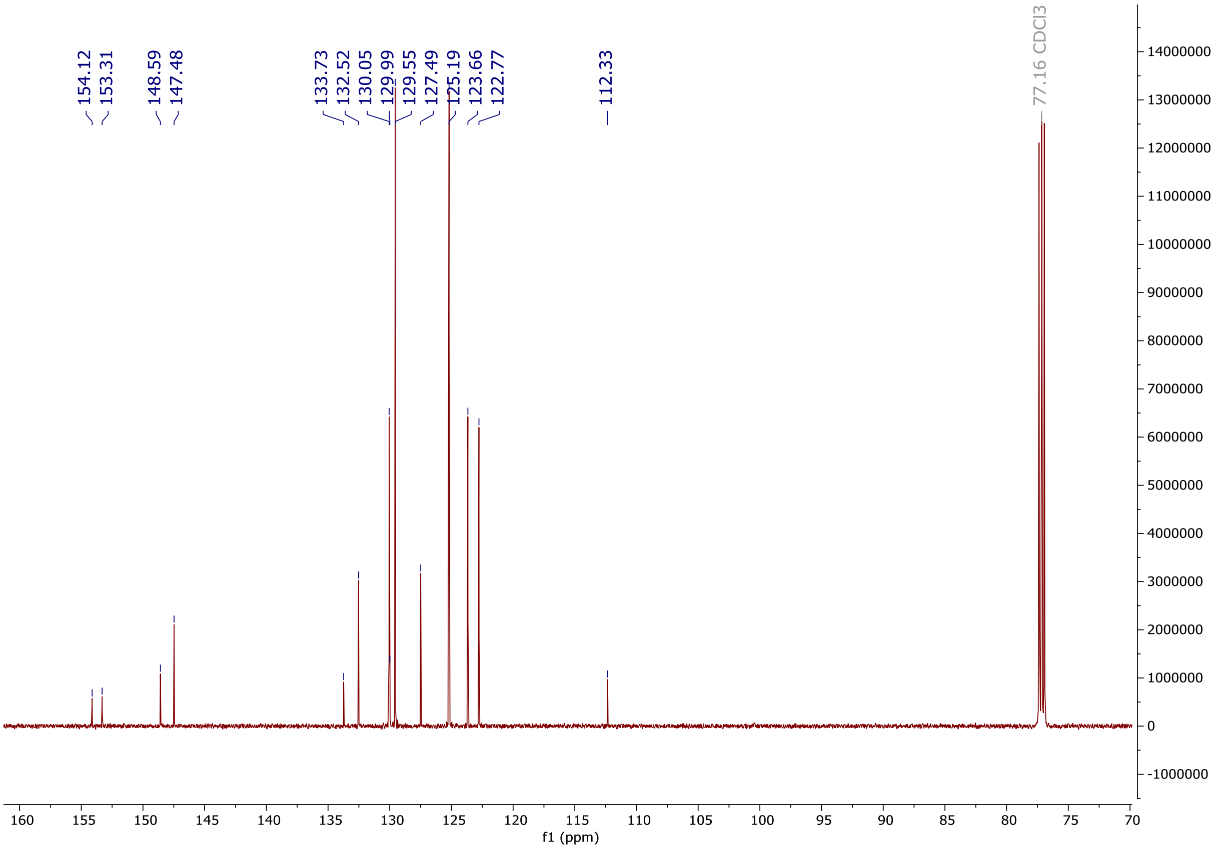

Figure S5. ^{13}C NMR of **A** in $CDCl_3$.

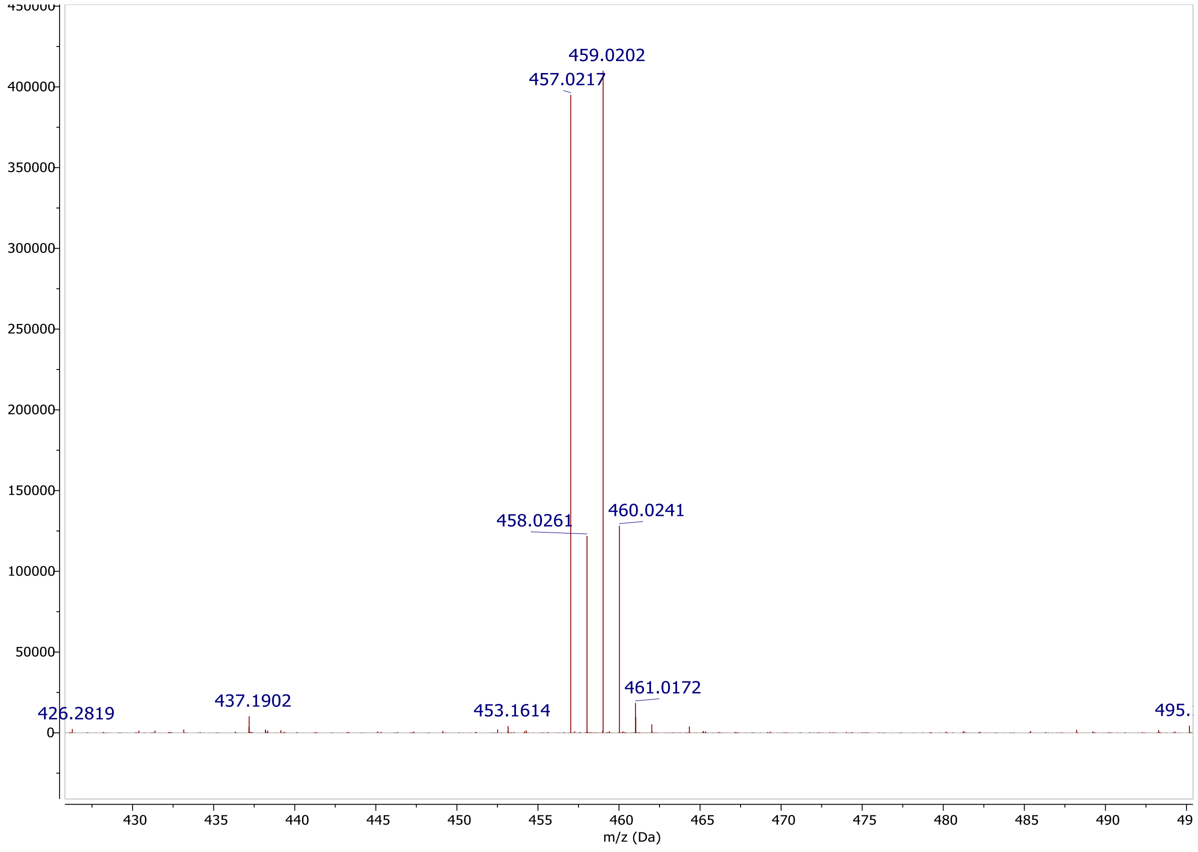

Figure S6. HR-MS of **A**.

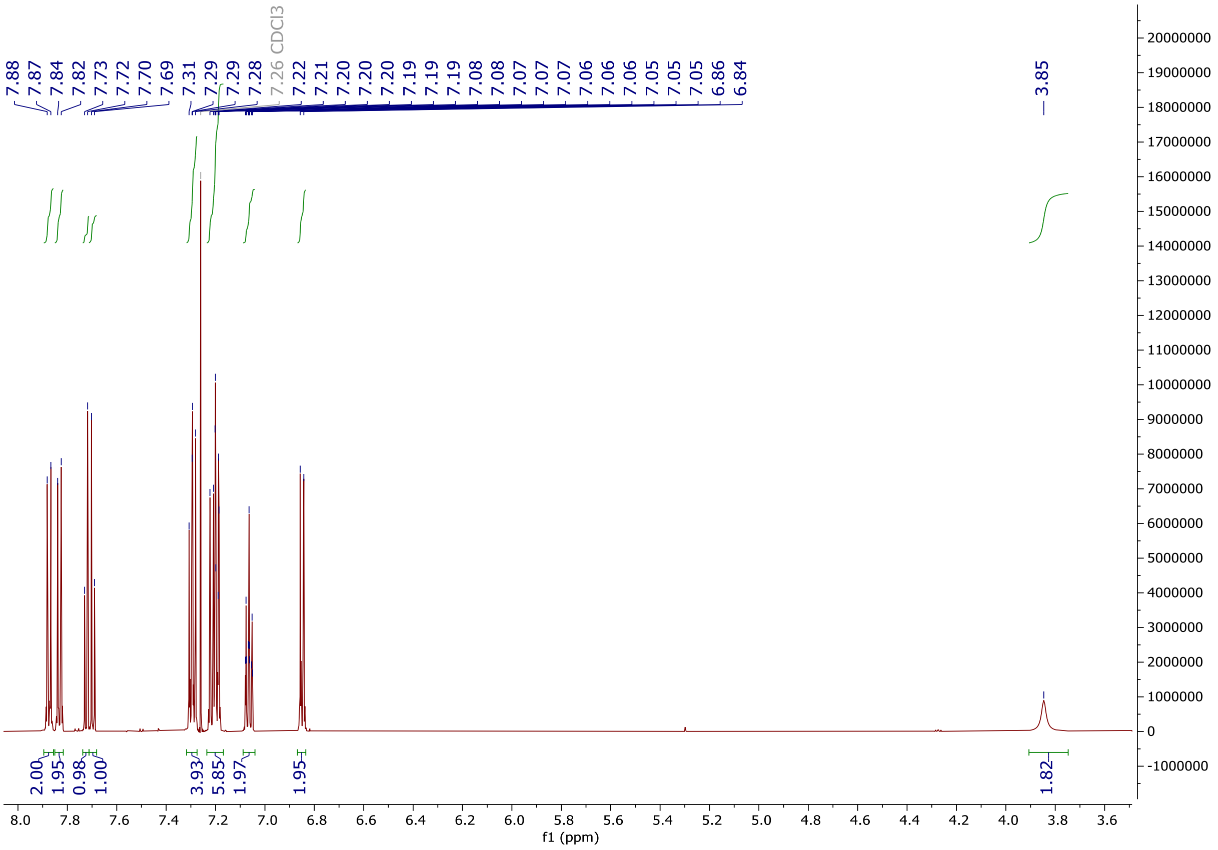

Figure S7. ^{1}H NMR of **B** in $CDCl_3$.

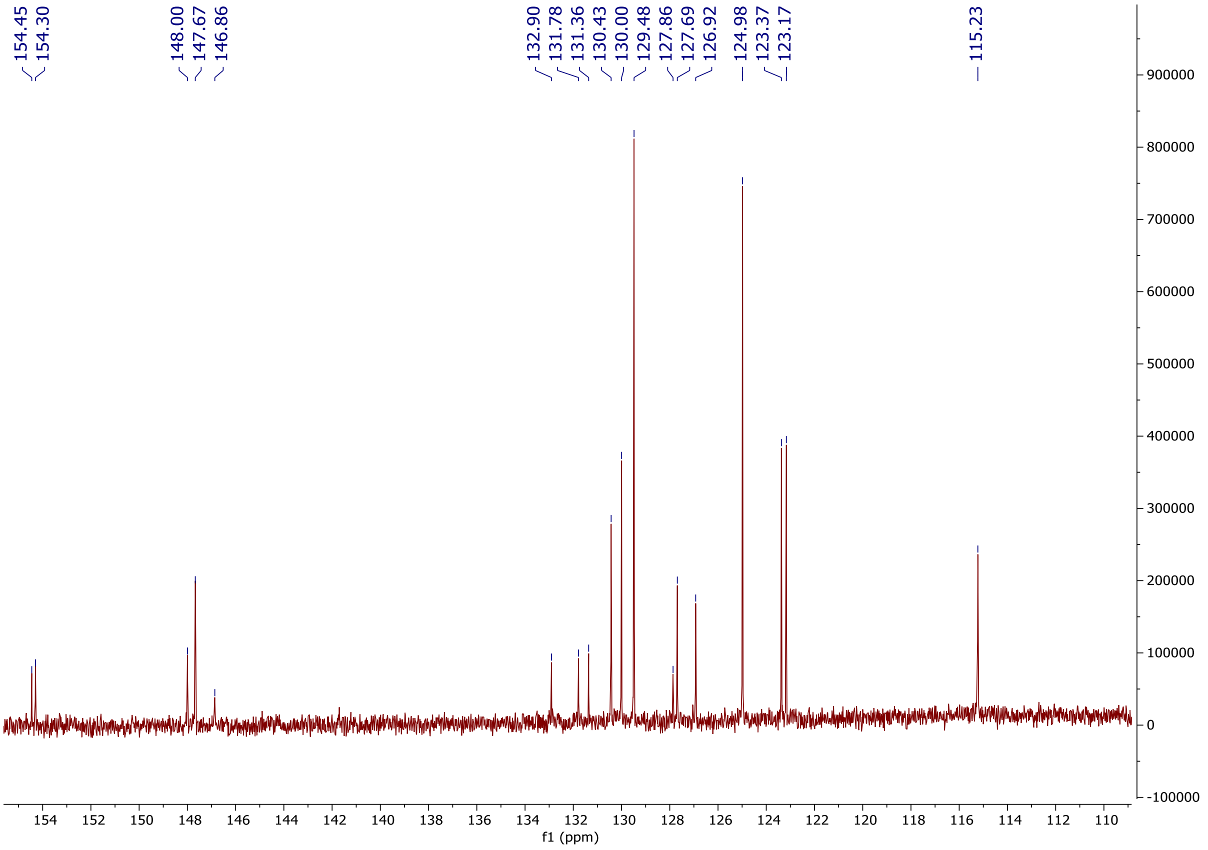

Figure S8. ^{13}C NMR of **B** in $CDCl_3$.

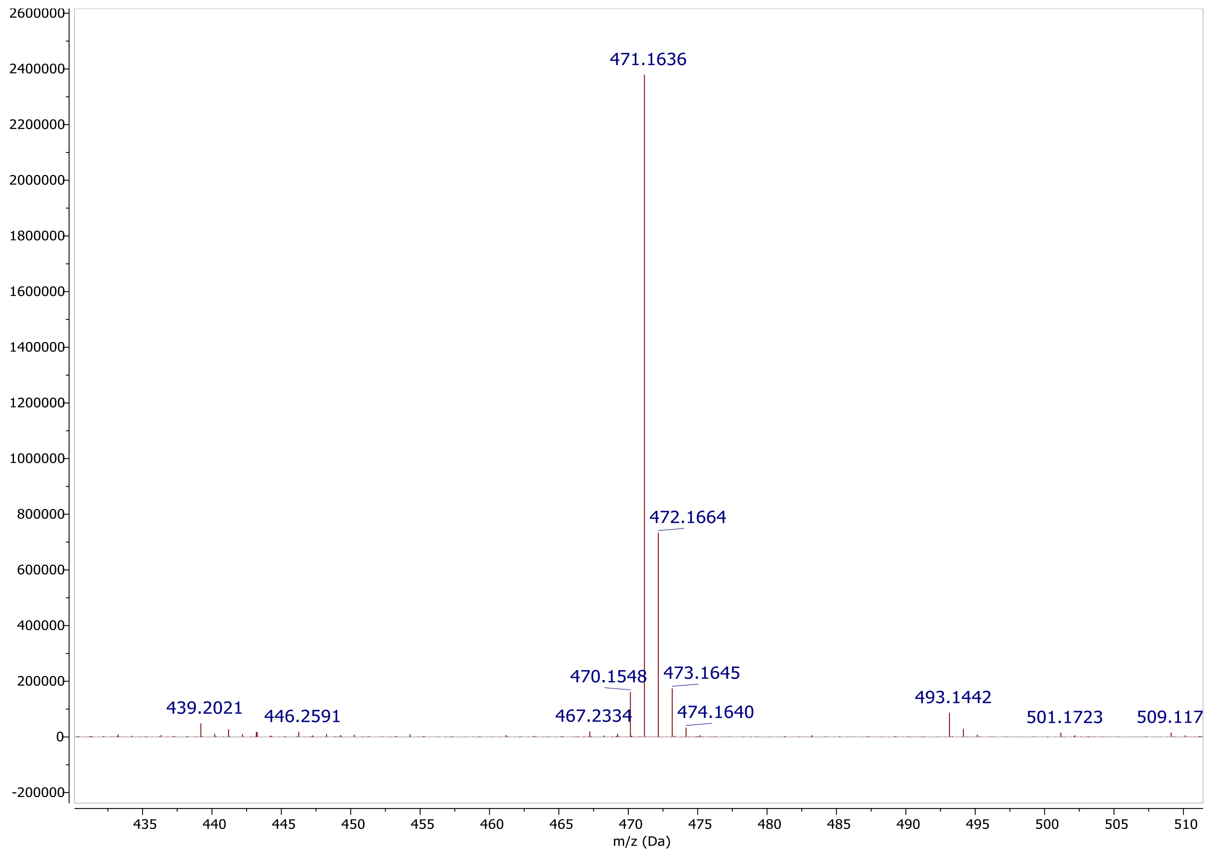

Figure S9. HR-MS of **B**.

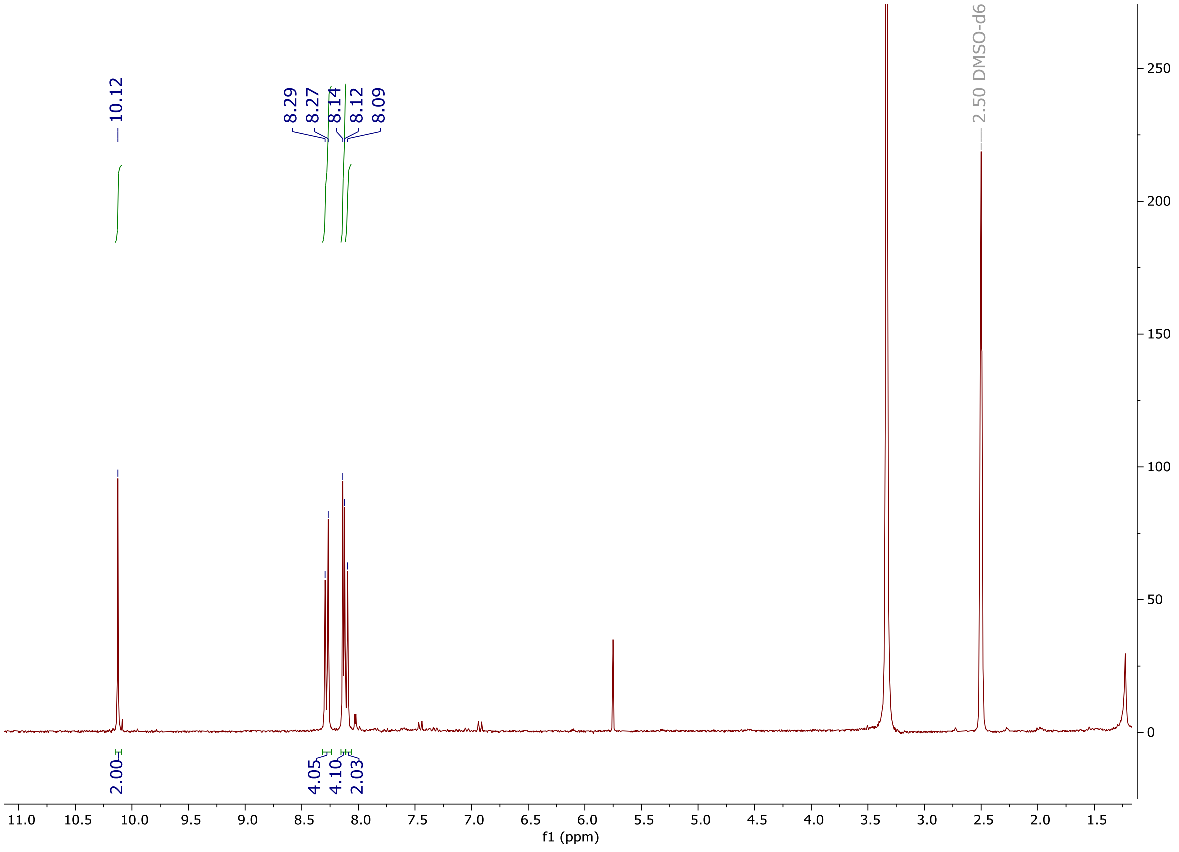

Figure S10. ^{1}H NMR of **C** in d_6-DMSO.

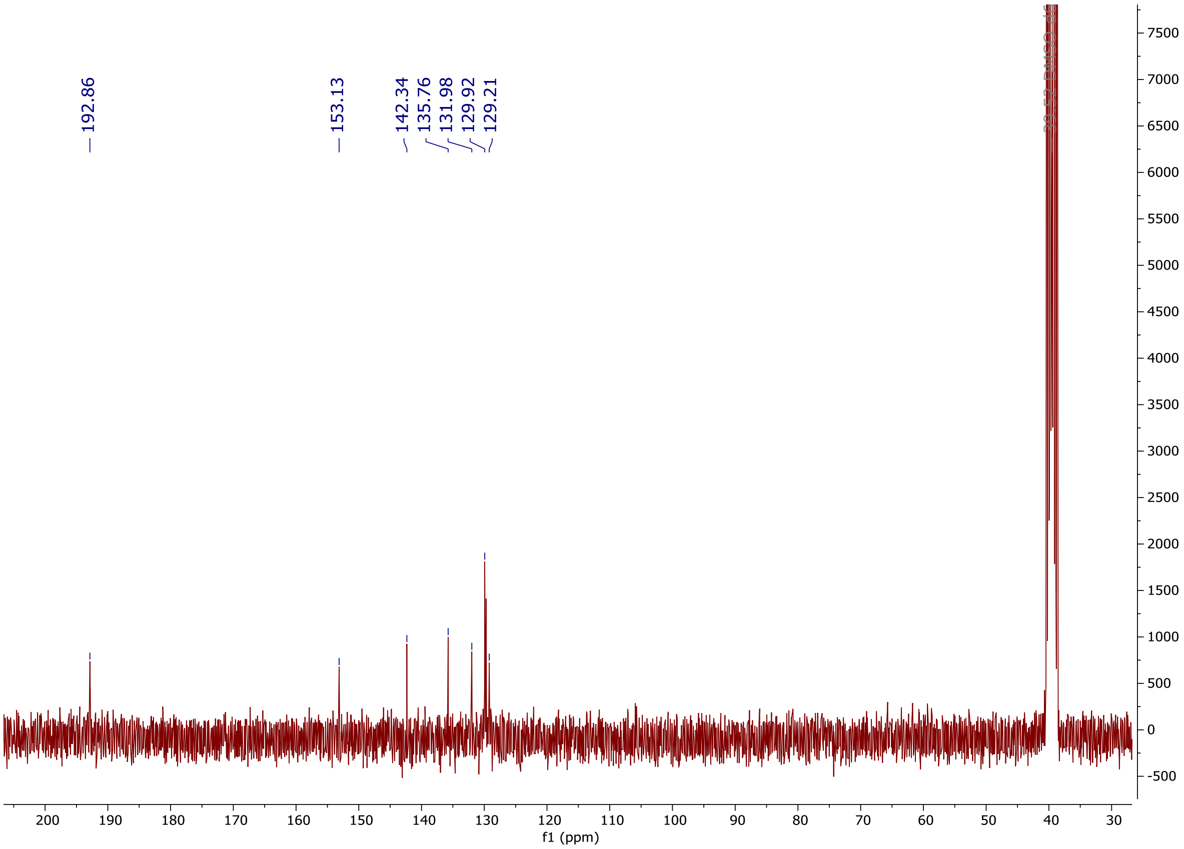

Figure S11. ^{13}C NMR of **C** in d_6-DMSO.

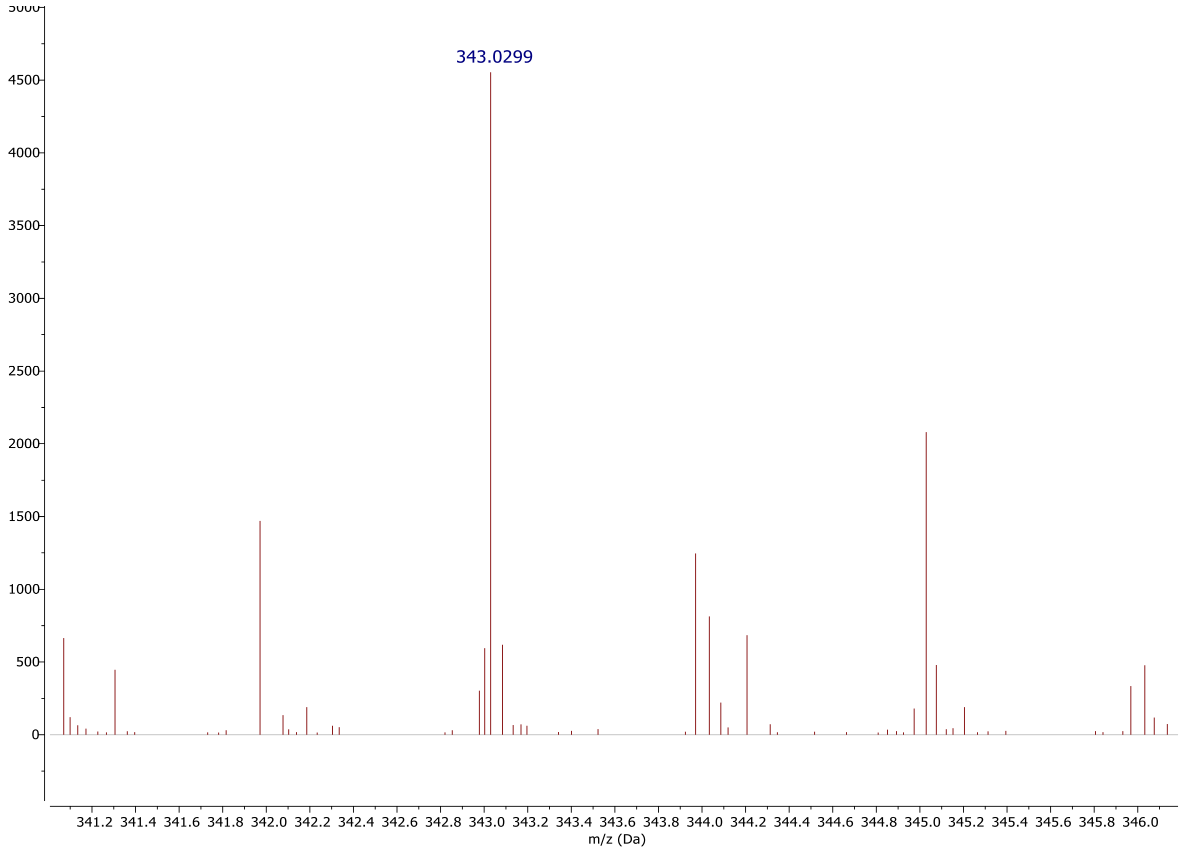

Figure S12. HR-MS spectra of **C**.

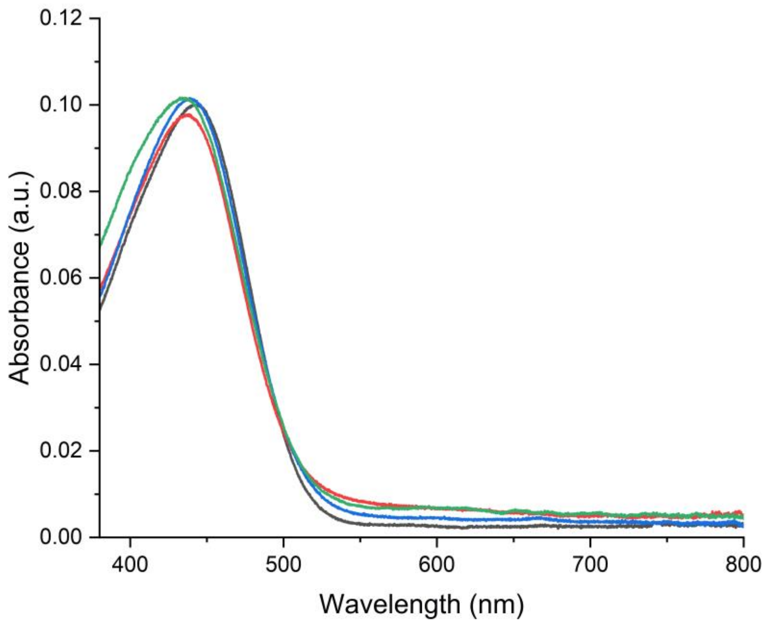

Figure S13. Absorption spectra of azomethine in toluene (black), dioxane (red), THF (blue) and dichloromethane (green).

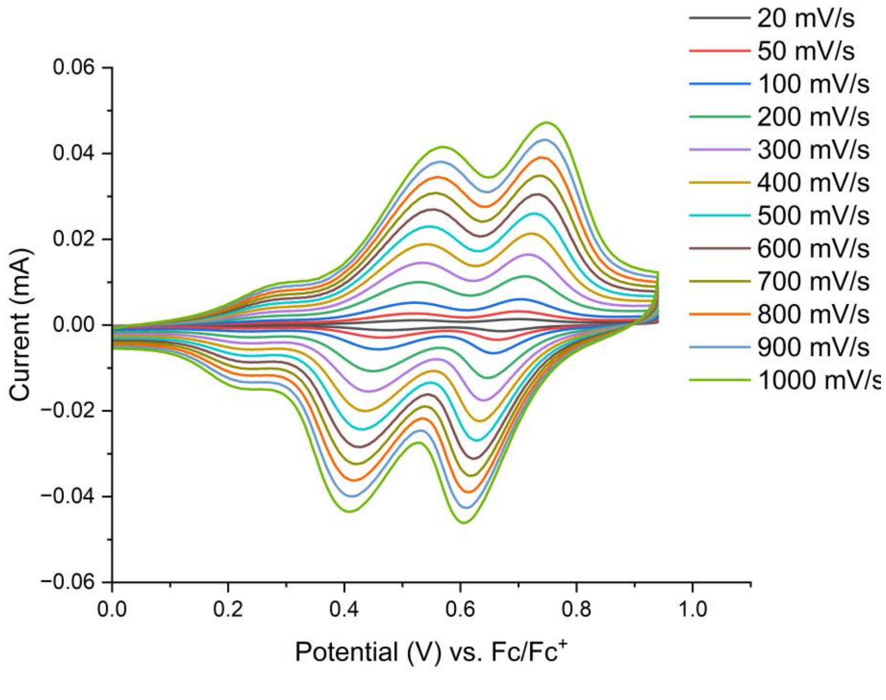

Figure S14. CV profiles of polyazomethine obtained at different scan rates.

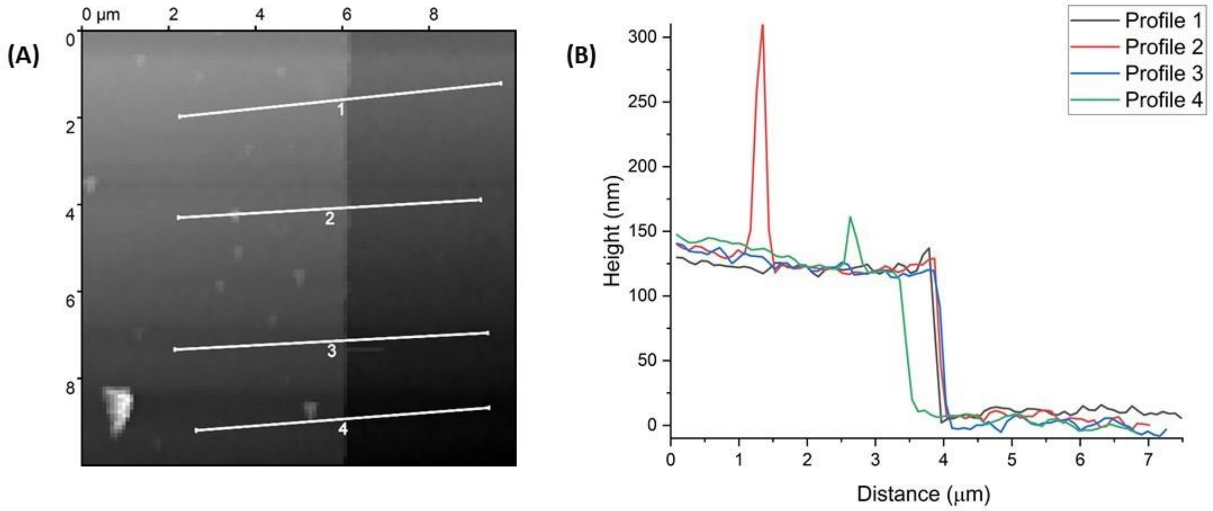

Figure S15. A) AFM micrograph of **poly-[TPA-(BTZ)$_3$-TPA]** deposited on ITO electrode showing the step between the ITO and the polymer surface. AFM cross-section profiles were measured at a marked places; B) AFM profiles measured at places shown in A).
